# Maximum common subgraph: some upper bound and lower bound results

**DOI:** 10.1186/1471-2105-7-S4-S6

**Published:** 2006-12-12

**Authors:** Xiuzhen Huang, Jing Lai, Steven F Jennings

**Affiliations:** 1 Department of Computer Science, Arkansas State University, State University, Arkansas 72467, USA; 2 Department of Applied Science, University of Arkansas at Little Rock, Little Rock, Arkansas 72204, USA; 3 Department of Information Science, University of Arkansas at Little Rock, Little Rock, Arkansas 72204, USA

## Abstract

**Background:**

Structure matching plays an important part in understanding the functional role of biological structures. Bioinformatics assists in this effort by reformulating this process into a problem of finding a maximum common subgraph between graphical representations of these structures. Among the many different variants of the maximum common subgraph problem, the *maximum common induced subgraph *of two graphs is of special interest.

**Results:**

Based on current research in the area of parameterized computation, we derive a new lower bound for the exact algorithms of the maximum common induced subgraph of two graphs which is the best currently known. Then we investigate the upper bound and design techniques for approaching this problem, specifically, reducing it to one of finding a maximum clique in the product graph of the two given graphs. Considering the upper bound result, the derived lower bound result is asymptotically tight.

**Conclusion:**

Parameterized computation is a viable approach with great potential for investigating many applications within bioinformatics, such as the maximum common subgraph problem studied in this paper. With an improved hardness result and the proposed approaches in this paper, future research can be focused on further exploration of efficient approaches for different variants of this problem within the constraints imposed by real applications.

## Background

### Introduction

Of the many challenging problems related to understanding the biological function of DNA, RNA, proteins, and metabolic and signalling pathways, one of the most important is comparing the structure of different molecules. The hypothesis is that structure determines function and therefore it should follow that molecules with similar structure should have similar function. Evaluating the similarity of structures can be reduced to a comparison of a set of abstracted graphs if the biological structures can be abstracted as graphs.

Using bioinformatic techniques, biological structure matching can be formulated as a problem of finding the *maximum common subgraph*. The solution to this problem has important practical applications in many areas of bioinformatics as well as in other areas, such as pattern recognition and image processing [1-3]. For example, protein threading, an effective method to predict protein tertiary structure [4-8], and RNA structural homology searching, a method for annotating and identifying new non-coding RNAs [9-12], both align a target structure against structure templates in a template database.

Song *et al *[13] makes the following definitions and proposes the following graphical models for RNA structural homology searching: A *structural unit *in a biopolymer sequence is a stretch of contiguous residues (nucleotides or amino acids). A *non-structural stretch *between two consecutive structural units is called a *loop*. A structure of the sequence is characterized by *interactions *among structural units. For example, structural units in a tertiary protein are α helices and β strands, called *cores*. Given a biopolymer sequence, a *structure graph *H = (V, E, A) can be defined such that each *vertex *in V(H) represents a structural unit, each *edge *in E(H) represents the interaction between two structural units, and each *arc *in A(H) represents the loop ended by two structural units. Similarly, the target sequence can also be represented as a *mixed graph *G, called a *sequence graph*. Based on the graphical representations, the structure-sequence alignment problem can be formulated as the problem of finding in the sequence graph G a subgraph *isomorphic *to the structure graph H such that the *objective function *optimizes the alignment score.

### Problem Definition

Throughout this paper, we will use the basic definitions and terminology from [1]: All graphs are simple, undirected graphs. Two graphs are *isomorphic *if there is a one-to-one correspondence between their vertices and there is an edge between two vertices in one graph if and only if there is an edge between the two corresponding vertices in the other graph. If edge (u, v) is an edge connecting u and v, then an *induced subgraph *G' of a graph G = (V, E) consists of a vertex subset V' ⊆ V and for all edges (u, v) ∈ E where u, v ∈ V'. A graph G_12 _is a *common induced subgraph *of two given graphs G_1 _and G_2 _if G_12 _is isomorphic to one induced subgraph G'_1 _of G_1 _as well as one induced subgraph G'_2 _of G_2_. A *maximum common induced subgraph *(MCIS) of two given graphs G_1 _and G_2 _is the common induced subgraph G_12 _with the maximum number of vertices. Similarly, the *maximum common edge subgraph *(MCES) is a subgraph with the maximum number of edges common to the two given graphs. The MCIS (or MCES) between two graphs can be further divided into a connected case and a disconnected case. All the different cases of the problem are useful within different biological contexts.

**Figure 1:**
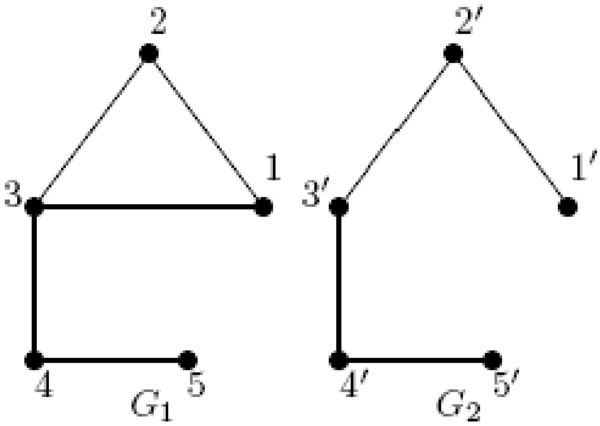
**MCIS of two graphs**. For G_1 _and G_2_, the maximum common induced subgraph of them contains four vertices, and the maximum common edge subgraph of them involves five vertices.

Figure 1 gives an illustration of MCIS of two graphs. In this figure, the maximum common induced subgraph of G_1 _and G_2 _contains four vertices (2, 3, 4 and 5) and the maximum common edge subgraph of them involves five vertices (1 through 5).

MCES can be transformed into a formulation of MCIS. Interested readers are referred to [1] for details of the transformation. Here we focus on the maximum common induced subgraph (MCIS) problem. For convenience, we call it the maximum common subgraph problem.

The maximum common subgraph problem is NP-complete [14] and therefore polynomial-time algorithms for it do not exist unless P = NP. In fact, the maximum common subgraph problem is APX-hard [15] which means that it has no constant ratio approximation algorithms. This problem is a famous combinatorial intractable problem. Approaches for the maximum common subgraph problem and different variants of this problem are intensively studied in the literature [1].

In this paper, we derive a strong lower bound result for the maximum common subgraph problem in the light of the current research progress in the research area of parameterized computation. We then design the approaches for addressing this problem.

## Methods

### Parameterized Computation and Recent Progress on Parameterized Intractability

Many problems with important real-world applications in life science are NP-hard within the context of the theory of NP-completeness. This excludes the possibility of solving them in polynomial time unless P = NP. For example, the problems of cleaning up data, aligning multiple sequences, finding the closest string, and identifying the maximum common substructure are all famous NP-hard problems in bioinformatics [16-18,1]. A number of approaches have been proposed in dealing with these NP-hard problems. For example, the highly-acclaimed approximation approach [19] tries to come up with a "good enough" solution in polynomial time instead of an optimal solution for an NP-hard optimization problem [20-23].

The theory of parameterized computation [17] is a newly developed approach introduced to address NP-hard problems with *small *parameters. It tries to give exact algorithms for an NP-hard problem when its natural parameter is small (even if the problem size is big). A *parameterized problem *Q is a decision problem consisting of instances of the form (x, k), where x is the problem description and the integer k = 0 is called the *parameter*. The parameterized problem Q is *fixed-parameter tractable *[17] if it can be solved in time f(k)|x|^O(1)^, where f is a recursive function. The class FPT contains all the problems that are fixed-parameter tractable. In this paper, we assume that complexity functions are "nice" with both the domain and range being non-negative integers and the values of the functions and their inverses are easily computed. For two functions f and g, we write f(n) = o(g(n)) if there is a nondecreasing and unbounded function λ such that f(n) = g(n)/λ(n). A function f is *subexponential *if f(n) = 2^O(n)^.

For a problem in the class FPT, research is focused on identifying more efficient, parameterized algorithms. There are many effective techniques to design parameterized algorithm including the methods of "bounded search tree" and "reduction to a problem kernel". Another example is the vertex cover problem.

#### Definition

Vertex cover problem: given a graph G and an integer k, determine if G has a vertex cover C of k vertices, i.e., a subset C of k vertices in G such that every edge in G has at least one endpoint in C. Here, the parameter is k.

Given a graph of n vertices, there is a parameterized algorithm that can solve the vertex cover problem in time O(kn + 1.286^k^) [24].

Accompanying the work on designing efficient and practical parameterized algorithms, a theory of parameter intractability has previously been developed [17]. In parameterized complexity, to classify fixed-parameter intractable problems, a hierarchy of classes (the W-hierarchy ∪_t = 0 _W [t], where W [t] ⊆ W [t+1] for all t = 0) have been introduced in which the 0-th level W [0] is the class *FPT*. The hardness and completeness have been defined for each level W [i] of the W-hierarchy for i = 1, and a large number of W [i]-hard parameterized problems have been identified [17]. For example, the clique problem is W[1]-hard.

#### Definition

Clique problem: given a graph G and an integer k, determine if G has a clique C of k vertices, i.e., a subset C of k vertices in G such that there is an edge in G between any two of these k vertices, i.e., the k vertices induce a complete subgraph of G. Here the parameter is k.

The clique problem can be solved in time O(n^k^), based on the enumeration of all the vertex subsets of size k for a given graph with n vertices.

It has become commonly accepted that no W[1]-hard (and W [i]-hard, i > 1) problem can be solved in time f(k)n^O(1) ^for any function f (i.e., W[1] ? FPT). W[1]-hardness has served as the hypothesis for fixed-parameter intractability. An example is a recent result by Papadimitriou and Yannakakis [25], showing that the database query evaluation problem is W[1]-hard. This provides strong evidence that the problem cannot be solved by an algorithm whose running time is of the form f(k)n^O(1)^, thus excluding the possibility of a practical algorithm for the problem even if the parameter k (the size of the query) is small as in most practical cases.

Based on the W[1]-hardness of the clique algorithm, computational intractability of problems in bioinformatics has been derived [26-31], the author point out that "Unless an unlikely collapse in the parameterized hierarchy occurs, the results proved in [31] that the problems longest common subsequence and shortest common supersequence are W[1]-hard rule out the existence of exact algorithms with running time f(k)n^O(1) ^(i.e., exponential only in k) for those problems. This does not mean that there are no algorithms with much better asymptotic time-complexity than the known O(n^k^) algorithms based on dynamic programming, e.g., algorithms with running time n^vk ^are not deemed impossible by our results."

Recent investigation has derived stronger computational lower bounds for well-known NP-hard parameterized problems [32,33]. For example, for the clique problem – which asks if a given graph of n vertices has a clique of size k – it is proved that unless an unlikely collapse occurs in parameterized complexity theory, the problem is not solvable in time f(k)n^o(k) ^for *any *function f. Note that this lower bound is asymptotically tight in the sense that the trivial algorithm that enumerates all subsets of k vertices in a given graph to test the existence of a clique of size k runs in time O(n^k^).

Based on the hardness of the clique problem, lower bound results for a number of bioinformatics problems have been derived [34]. For example, our results for the problem's longest common subsequence and shortest common supersequence have strengthened the results in [31] significantly and advanced the understanding on the complexity of the problems. We show that it is actually unlikely that the problems can be solved in time n^γ(k) ^for *any *sublinear function γ(k) and the known dynamic programming algorithms of running time O(n^k^) for the problems are actually asymptotically optimal.

In the following section, we derive the lower bound for exact algorithms of the maximum common subgraph problem.

### Lower Bound for Maximum Common Subgraph Problem

The formal parameterized version of the maximum common subgraph problem is described above; we choose the number of vertices in the common subgraph as the parameter. Based on the reduction from the parameterized clique problem to the parameterized common subgraph problem, we derive the hardness result of the parameterized common subgraph problem.

An NP optimization problem Q is a four-tuple (I_Q_, S_Q_, f_Q_, opt_Q_) [19], where:

1. I_Q _is the set of input instances. It is recognizable in polynomial time;

2. For each instance x ∈ I_Q_, S_Q_(x) is the set of feasible solutions for x, which is defined by a polynomial p and a polynomial time computable predicate π (p and π only depend on Q); S_Q_(x) = {y: |y| = p(|x|) and π(x, y)};

3. f_Q_(x, y) is the objective function mapping a pair x ∈ I_Q _and y ∈ S_Q_(x) to a non-negative integer; the function f_Q _is computable in polynomial time;

4. opt_Q_∈ {max, min}. Q is called a *maximization problem *if opt_Q _= max and a *minimization problem *if opt_Q _= min.

An NP optimization problem Q can be parameterized in a natural way as follows [35,32]:

#### Definition

Let Q = (I_Q_, S_Q_, f_Q_, opt_Q_) be an NP optimization problem. The *parameterized version *of Q is defined as:

1. If Q is a maximization problem, then the parameterized version of Q is defined as Q = {(x, k) | x ∈ I_Q _^ opt_Q(x) _= k };

2. If Q is a minimization problem, then the parameterized version of Q is defined as Q = {(x, k) | x ∈ I_Q _^ opt_Q(x) _= k}.

We now provide the definitions of the maximum common subgraph problem and the parameterized common subgraph problem.

#### Definition

Maximum common subgraph problem:

Input: two graphs G_1 _= (V_1_, E_2_) and G_2_= (V_2_, E_2_).

Output: the maximum common vertex-induced subgraph of the two graphs G_1 _and G_2_.

#### Definition

Parameterized common subgraph problem:

Input: two graphs G_1 _= (V_1_, E_2_) and G_2_= (V_2_, E_2_), and a positive integer k;

Parameter: k;

Output: "Yes", if there is a common vertex-induced subgraph of k vertices, i.e., a common subgraph of size k of the two graphs G_1 _and G_2_. Otherwise, output "No".

#### Lemma 1

The parameterized common subgraph problem is W[1]-hard.

Proof: We will give an FPT-reduction from clique to the parameterized common subgraph problem as follows.

Given an instance (G, k) of the clique problem, where the graph G has n vertices and k is a positive integer, we construct an instance of the parameterized common subgraph problem as follows: let G_1 _be the graph G, and G_2 _a complete graph of k vertices. The problem can therefore be stated as "Is a common vertex-induced subgraph of k vertices for the graphs G_1 _and G_2_?"

We can verify that the graph G has a clique of size k if and only if the graphs G_1 _and G_2 _have a common subgraph of k vertices. Since the reduction may be finished in polynomial time O(nk), the reduction is an FPT-reduction from clique to parameterized common subgraph problem.

To prove our main result, we will use the definition of linear FPT-reduction and W_1_[1]-hard [36]:

#### Definition

A parameterized problem Q is *linear FPT-reducible*, or more precisely, *FPT*_*l*_*-reducible*, to a parameterized problem Q' if there exist a function f and an algorithm A of running time f(k)n^O(1) ^that, on each (k, n)-instance x of Q, produces a (k', n')-instance x' of Q', where k' = O(k), n' = n^O(1)^, and x is a yes-instance of Q if and only if x' is a yes-instance of Q'.

Linear FPT-reduction has the transitivity property [36,34]. The transitivity of the FPT_l_-reduction is proved in the following lemma:

#### Lemma 2

Let Q_1_, Q_2 _and Q_3 _be three parameterized problems. If Q_1 _is FPT_l_-reducible to Q_2_, and Q_2 _is FPT_l_-reducible to Q_3_, then Q_1 _is FPT_l_-reducible to Q_3_.

Proof: If Q_1 _is FPT_l_-reducible to Q_2_, then there exists a function f_1 _and an algorithm A_1 _of running time f_1_(k_1_)n_1_^o(k1)^m_1_^O(1)^, such that for each (k_1_, n_1_, m_1_)-instance x_1 _of Q_1_, the algorithm A_1 _produces a (k_2_, n_2_, m_2_)-instance x_2 _of Q_2_, where n_2 _= n_1_^O(1)^, m_2 _= m_1_^O(1)^, and k_2 _= c_1_k_1_, where c_1 _is a constant.

If Q_2 _is FPT_l_-reducible to Q_3_, then there exists a function f_2 _and an algorithm A_2 _of running time f_2_(k_2_)n_2_^O(k2) ^m_2_^O(1)^, such that on each (k_2_, n_2_, m_2_)-instance x_2 _of Q_2_, the algorithm A_2 _produces a (k_3_, n_3_, m_3_)-instance x_3 _of Q_3_, where k_3 _= O(k_2_), n_3 _= n_2_^O(1)^, m_3 _= m_2_^O(1)^.

We now have an algorithm A that reduces Q_1 _to Q_3_, as follows: For a given (k_1_, n_1_, m_1_)-instance x_1 _of Q_1_, A first calls the algorithm A_1 _on x_1 _to construct a (k_2_, n_2_, m_2_)-instance x_2 _of Q_2_, where k_2 _= c_1_k_1_, n_2 _= n_1_^O(1)^, and m_2 _= m_1_^O(1)^. Then A calls the algorithm A_2 _on x_2 _to construct a (k_3_, n_3_, m_3_)-instance x_3 _of Q_3_. It is therefore obvious that x_3 _is a yes-instance of Q_3 _if and only if x_1 _is a yes-instance of Q_1_. Moreover, from k_2 _= c_1_k_1 _and k_3 _= O(k_2_), we have k_3 _= O(k_1_), and from n_2 _= n_1_^O(1)^, m_2 _= m_1_^O(1)^, n_3 _= n_2_^O(1)^, m_3 _= m_2_^O(1)^, we get n_3 _= n_1_^O(1) ^and m_3 _= m_1_^O(1)^. Finally, since the invocation of algorithm A_1 _on x_1 _takes time f_1_(k_1_)n_1_^o(k1) ^m_1_^O(1)^, the invocation of algorithm A_2 _on x_2 _takes time f_2_(k_2_)n_2_^O(k2) ^m_2_^O(1)^, k_2 _= c_1_k_1_, n_2 _= n_1_^O(1)^, and m_2 _= m_1_^O(1)^, we conclude that the running time of algorithm A is bounded by f_1_(k_1_)n_1_^O(k1) ^m_1_^O(1)^, where f(k_1_) = f_1_(k_1_) + f_2_(c_1_k_1_). By definition, A is an FPT_l_-reduction from Q_1 _to Q_3_; i.e., Q_1 _is FPT_l_-reducible to Q_3_.

#### Definition

A parameterized problem Q is W[1]-hard under the FPT_l_-reduction, or more precisely W_l_[1]-hard, if the *Weighted antimonotone CNF 2SAT *(abbreviated wcnf -2sat^-^) problem is FPT_l_-reducible to Q.

In particular, it has been shown [32,33] that the clique problem is W_l_[1]-hard.

#### Lemma 3

(From theorem 5.2 of [33]) Unless all SNP problems are solvable in subexponential time, no W_l_[1]-hard problem can be solved in time f(k)n^O(k) ^for any recursive function f.

Note Papadimitriou and Yannakakis [30] have introduced the class SNP which contains many well-known NP-hard problems. Some of these problems have been the major targets in the study of exact algorithms, but have so far resisted all efforts for the development of subexponential time algorithms to solve them. Thus, it has been commonly agreed that it is unlikely that all SNP problems are solvable in subexponential time. A recent result showed the equivalence between the statement that "all SNP problems are solvable in subexponential time" and the collapse of a parameterized class called Mini[1,37] to FPT, which is also considered as an unlikely collapse in parameterized computation.

#### Lemma 4

The parameterized common subgraph problem is W_l_[1]-hard.

Proof: Referring to the proof of Lemma 1, the reduction from a clique to a parameterized common subgragh problem is a linear FPT-reduction.

Based on the transitivity property of the linear FPT-reduction of Lemma 2, and the fact that the clique problem is W_l_[1]-hard, the parameterized common subgraph problem could not be solved in time f(k)n^O(k)^, where k is the number of vertices in the common subgraph and f is any recursive function, unless some unlikely collapse (Mini[1] = FPT) occurs in parameterized computation.

From Lemma 4 and Proposition 3, we have the following theorem:

#### Theorem

Given two graphs G_1 _and G_2 _with each graph having n vertices, there is no algorithm of time f(k)n^O(k) ^for the parameterized common subgraph problem, where k is the number of vertices in the common subgraph and f is any recursive function, unless some unlikely collapse (Mini[1] = FPT) occurs in parameterized computation.

In consideration of the upper-bound result, we now show that our lower-bound result for the maximum common subgraph problem presented here is asymptotically tight.

### Upper Bound – Clique Based Approaches

The following approach for the maximum common subgraph problem is based on the reduction [15,1] from a maximum common subgraph problem to the maximum clique problem.

From two graphs G_1_= (V_1_, E_1_) and G_2_= (V_2_, E_2_), a new graph G= (V, E) is derived as follows: Let V = V_1 _× V_2 _and call V a set of pairs. Call two pairs <u_1_, u_2_> and <v_1_, v_2_> compatible if u_1 _≠ v_1 _and u_2 _≠ v_2 _and if they preserve the edge relation, that is, there is an edge between u_1 _and v_1 _if and only if there is an edge between u_2 _and v_2_. Let E be the set of compatible edges. A k-clique in the new graph G can be interpreted as a matching between two induced k-node subgraphs. The two subgraphs are isomorphic since the compatible pairs preserve the edge relations. The new graph G is called the modular product graph of the two graphs G_1 _and G_2_.

We suppose n = |V_1_| = |V_2_| (The analysis for the case when |V_1_| ? |V_2_|, is similar, and thus is omitted). From the construction of G, we have |V| = n^2^. By a close observation of the new graph G, we can see that G is indeed an n-partite graph, where the vertices are partitioned into n disjoint partitions with each partition having n vertices.

We may use a matrix to denote the n^2 ^vertices of the n-partite graph with n vertices in each partition.

v_{1,1}_, v_{1,2}_, ..., v_{1,n}_

v_{2,1}_, v_{2,2}_, ..., v_{2,n}_

... ...

v_{n,1}_, v_{n,1}_, ..., v_{n,n}_

The n vertices of the first row v_{1,i}_, 1 = i = n, belong to partition one of the n-partite graph. The n vertices of the second row v_{2,i}_, 1 = i = n, belong to partition two and so on.

There is no edge between any two vertices within the same partition. Edges only appear between two vertices that are in two different partitions. So, at most one vertex from each partition (of the n vertices) could be in a clique of the graph. Therefore, to find a clique of size k, there will be n^k ^possible ways for choosing the clique vertices. For each possible way, the algorithm needs O(k^2^) time to check if it constructs a clique of size k. Therefore, this gives an algorithm of time O(n^k^k^2^) for the maximum common subgraph problem. We call this algorithm ALG-COMMON SUBGRAPH for the convenience of the following discussion.

This problem – when the maximum clique size k is equal to n – has been studied by Sze *et al *[38]:

#### Definition

Given an n-partite graph G with n vertices in each part, the n-CLIQUE_np _problem finds an n-clique in the graph G.

For this problem, they developed a fast and exact divide-and-conquer approach. The basic idea of this novel approach is to subdivide the given n-partite graph into several n_0_-partite subgraphs with n_0 _< n and solve each smaller subproblem independently using a branch-and-bound approach as long as the number of cliques of size n_0 _in each subproblem is not too high. The reader is referred to [38] for the details of this divide-and-conquer approach. However, their approach in the worst case still has the same upper bound.

Given this O(n^k ^k^2^)-time algorithm for the maximum common subgraph problem, the lower bound result of our Theorem is asymptotically tight.

When the number of vertices in the common subgraph k is not very far away from the value of n, we define k = n – c, where c is a constant. We illustrate the basic idea for c = 1 as follows [39]: Suppose the n-partite graph G has a clique C of size k-1. We add one more vertex to each of the n partitions. And we also add edges from this vertex to any vertices (except the newly added vertices) that are not in the same partition. Now we get a new graph G'. G' is an n-partite graph with n + 1 vertices in each partition. The new graph G' has a clique C' of size n if and only if the original n-partite graph G has a clique of size (n-1). The vertices of this clique C' include the vertices of the original clique C and one newly added vertex.

For the newly constructed graph G', we can now apply the algorithm ALG-COMMON SUBGRAPH without any change. And we need time O((n+1)^n ^n^2^). After we find the clique C', we just remove the newly added vertex and return the other vertices of C'.

Similarly, if the n-partite graph G has a clique of size k – c, where c is a positive integer constant, we can find the clique by adding c new vertices and associated edges as described above and then applying the algorithm ALG-COMMON SUBGRAPH which runs in time O((n+c)^n ^n^2^).

This simple idea of dealing with cliques of a size less than n is useful since it makes the algorithm ALG-COMMON SUBGRAPH work uniformly for finding cliques of different sizes on n-partite graphs. In the following, we give the following algorithm for finding cliques of size k – c.

#### Algorithm for (K-C)-CLIQUE

INPUT: an n-partite graph G, with n vertices in each partition, and a small constant c, where c is a positive integer;

OUTPUT: a clique of size no less than k – c;

Step 1: For i = 0 to c do

• Step 1.1: Construct a new graph G_1_, by adding i new vertices to each partition of the graph G and adding edges from each of the new vertices to any vertices (except the newly added vertices) that are not in the same partition.

• Step 1.2: Apply the algorithm ALG-COMMON SUBGRAPH on the graph G_1_.

• Step 1.3: If a clique C_1 _is found, then return "a clique C of size k – i has been found" (C is constructed by removing all the newly-added vertices from the clique C_1_).

• Endfor

Step 2: Return "no clique has been found".

We now propose two approaches for the maximum common subgraph problem which are based on the relationship between the vertex cover problem and the clique problem:

#### Algorithm 1: ALG-APPROX-CLIQUE

INPUT: an n-partite graph G, with n vertices in each partition, and a small constant c, where c is a positive integer;

OUTPUT: a clique for the graph G.

Step 1. Compute the complement graph G' of the modular product graph G = (V, E) of graph G_1 _and G_2_;

Step 2. Apply the approximation algorithm for the vertex cover problem to get a vertex cover C;

Step 3. Return V – C as the clique vertex set.

ALG-APPROX-CLIQUE gives an approximate solution for the maximum common subgraph problem in polynomial time. This approach uses the following approximation algorithm for the vertex cover problem with an approximation ratio 2 in [40]:

#### ALG-APPROX-VERTEX COVER

INPUT: a graph G = (V, E);

OUTPUT: a vertex cover C of approximation ratio 2 for the graph G.

Step 1. C ← Φ;

Step 2. E' ← E(G);

Step 3. While E' ≠ Φ

• Step 3.1. Let (u, v) be an arbitrary edge of E';

• Step 3.2. C = C ∪ {u, v};

• Step 3.3. Remove from E' every edge incident on either u or v;

Step 4. Return C as the vertex cover set.

In this algorithm, ALG-APPROX-VERTEX COVER selects an edge from the set of edges of the graph G = (V, E) and adds it to C. Repeating this procedure for (u, v) ∈ E(G) and deleting edges from E' that are covered by u or v results in a running time of O(V+E).

#### Algorithm 2: ALG-EXACT-MAXCLIQUE

INPUT: an n-partite graph G, with n vertices in each partition, and a small constant c;

OUTPUT: a clique for the graph G.

Step 1. Compute the complement graph G' of the modular product graph G = (V, E) of graph G_1 _and G_2_;

Step 2. Apply the parameterized exact algorithm for the Vertex Cover problem on G' and compute the minimum vertex cover C_0_.

Step 3. Return the maximum clique with the vertex set V – C_0_.

Alternatively, ALG-EXACT-MAXCLIQUE could apply in Step 2 the current best algorithm for vertex cover [24] which is of time O(kn + 1.286^k^). By running the vertex cover algorithm for at most n times, we produce the minimum vertex cover of the product graph G.

## Results

In this paper we investigated the lower-bound result for the maximum common subgraph problem. We proved that it is unlikely that there is an algorithm of time f(k)n^O(k) ^for the problem, where k is the number of vertices in the common subgraph and f is any recursive function. We then presented the upper bound of algorithms which solve this problem: O(n^k^k^2^) time where k is the number of vertices in the common subgraph. In consideration of the upper-bound result, we point out that our lower-bound result for the maximum common subgraph problem is asymptotically tight.

## Conclusion

Parameterized computation is a viable approach with great potential for investigating many applications within bioinformatics, such as the maximum common subgraph problem studied in this paper. With an improved hardness result and the proposed approaches in this paper, future research can be focused on further exploration of efficient approaches for different variants of this problem within the constraints imposed by real applications.

## Authors' contributions

XH carried out the study on the lower bound and approaches for the maximum common subgraph problem and helped to provide background information on parameterized computation theory. JL and SFJ participated in the design and expression of the algorithms for the maximum common subgraph problem. All authors have read and approved the final manuscript.
